# Genetic alterations in peritoneal metastatic tumors predicted the outcomes for hyperthermic intraperitoneal chemotherapy

**DOI:** 10.3389/fonc.2023.1054406

**Published:** 2023-04-26

**Authors:** Quynh-Anh Nguyen, Wan-Hsuan Chou, Mao-Chih Hsieh, Che-Mai Chang, Wei-Tzu Luo, Yu-Ting Tai, Wei-Chiao Chang

**Affiliations:** ^1^ Department of Clinical Pharmacy, School of Pharmacy, Taipei Medical University, Taipei, Taiwan; ^2^ Department of General Surgery, Wan Fang Hospital, Taipei Medical University, Taipei, Taiwan; ^3^ Department of Anesthesiology, School of Medicine, College of Medicine, Taipei Medical University, Taipei, Taiwan; ^4^ Department of Anesthesiology, Wan Fang Hospital, Taipei Medical University, Taipei, Taiwan; ^5^ Master Program in Clinical Genomics and Proteomics, School of Pharmacy, Taipei Medical University, Taipei, Taiwan; ^6^ Department of Pharmacy, Wan Fang Hospital, Taipei Medical University, Taipei, Taiwan; ^7^ Integrative Research Center for Critical Care, Wan Fang Hospital, Taipei Medical University, Taipei, Taiwan

**Keywords:** hyperthermic intraperitoneal chemotherapy (HIPEC), peritoneal metastasis, whole exome sequencing (WES), somatic mutation, AGAP5

## Abstract

**Introduction:**

Cytoreductive surgery (CRS) and hyperthermic intraperitoneal chemotherapy (HIPEC) are considered for patients with peritoneal metastasis (PM). However, patients selection that relies on conventional prognostic factors is not yet optimal. In this study, we performed whole exome sequencing (WES) to establish tumor molecular characteristics and expect to identify prognosis profiles for PM management.

**Methods:**

In this study, blood and tumor samples were collected from patients with PM before HIPEC. Tumor molecular signatures were determined using WES. Patient cohort was divided into responders and non-responders according to 12-month progression-free survival (PFS). Genomic characteristics between the two cohorts were compared to study potential targets.

**Results:**

In total, 15 patients with PM were enrolled in this study. Driver genes and enriched pathways were identified from WES results. AGAP5 mutation was found in all responders. This mutation was significantly associated with better OS (p = 0.00652).

**Conclusions:**

We identified prognostic markers that might be useful to facilitate decision-making before CRS/HIPEC.

## 1 Introduction

Peritoneal metastases (PMs) are aggressive advanced-stage manifestations that predominantly originate from intra-abdominal malignancies such as gastric, colorectal, appendiceal, and ovarian cancers. Patients with PM showed limited overall survival (OS) of approximately 3.1 months (1). Cytoreductive surgery and systemic chemotherapy are still the standard treatments. Over the last two decades, marginal efficacy of systemic palliative chemotherapy in PM management (2–4) has led to an alternative approach: cytoreductive surgery (CRS) with hyperthermic intraperitoneal chemotherapy (HIPEC) (5).

Poor distribution of systemic chemotherapy to the peritoneal cavity is due to the plasma-peritoneal barrier and high interstitial pressure of tumors. Therefore, intraperitoneal infusion offers preferentially higher efficacy in delivering chemotherapeutic drugs to tumor nodules while localizing the diffusion of these drugs in the peritoneum to decrease their systemic toxicity (5, 6). Furthermore, hyperthermia with the range of temperature from 41 to 43°C augments the cytotoxic effect of intraperitoneal chemotherapeutic agents *via* DNA repair inhibition, promotion of heat shock proteins, immune cell recruitment (e.g., natural killer cells, dendritic cells, T cells) and apoptosis (7, 8).

While systemic chemotherapy increases the median OS of patients with PM to roughly 1 year (9-15 months) (6, 9, 10), some results from CRS/HIPEC have shown superior efficacy to improve the survival duration. Median survival times varied according to the origin of the primary cancer. For colorectal origin, the median OS of PM patients receiving CRS/HIPEC was 32-41 months (11, 12); patients with epithelial ovarian cancer achieved a median OS of 45.7 months (13); and the duration of survival for patients with mesothelioma was up to 53 months (14).

Despite these promising results, a considerable group of patients were reported to have relapsed within 12 months following CRS/HIPEC (15–17). In addition, recent randomized clinical trials failed to confirm the favorable outcome of CRS/HIPEC over mono-modal systemic chemotherapy or palliative surgery in colorectal PM and recurrent epithelial ovarian cancer (18, 19). The controversial results highlighted shortcomings of current guidelines for CRS/HIPEC. First, the protocols vary across institutes regarding open or closed abdominal perfusion, chemotherapeutic drugs used, administration duration, and the optimal temperature range, making it difficult to extrapolate results to all patients (20, 21). Second, conventional prognostic factors for CRS/HIPEC prominently depend on clinical and pathological data such as patient’s clinical performance status (Eastern Cooperative Oncology Group (ECOG) score, peritoneal carcinomatosis index (PCI), histologic tumor grade, lymph node status, signet ring cell differentiation, the completeness of cytoreduction, and the experience of the operative team (22, 23). Nonetheless, the biological heterogeneity of peritoneal tumors in different patients raises concerns that the generally standardized regimen of HIPEC might not be effective in all cases and plausibly necessitates a personalized approach to optimize clinical decision-making. Thus, insights of tumor molecular characteristics are of great value to improve patient selection for CRS/HIPEC.

Recently, high-throughput sequencing technology has shed light on genomic profiles in individual cancer therapy. Indeed, genomic application for prognostic significance after CRS/HIPEC therapy was reported (24–26). However, the current information is still not able to be translated into clinical applications (27). Herein, we sought to unravel the tumor-specific genetic alterations, thereby providing predictive genetic-guided factors associated with HIPEC responsiveness.

## 2 Materials and methods

### 2.1 Patient cohort

**Figure 1:**
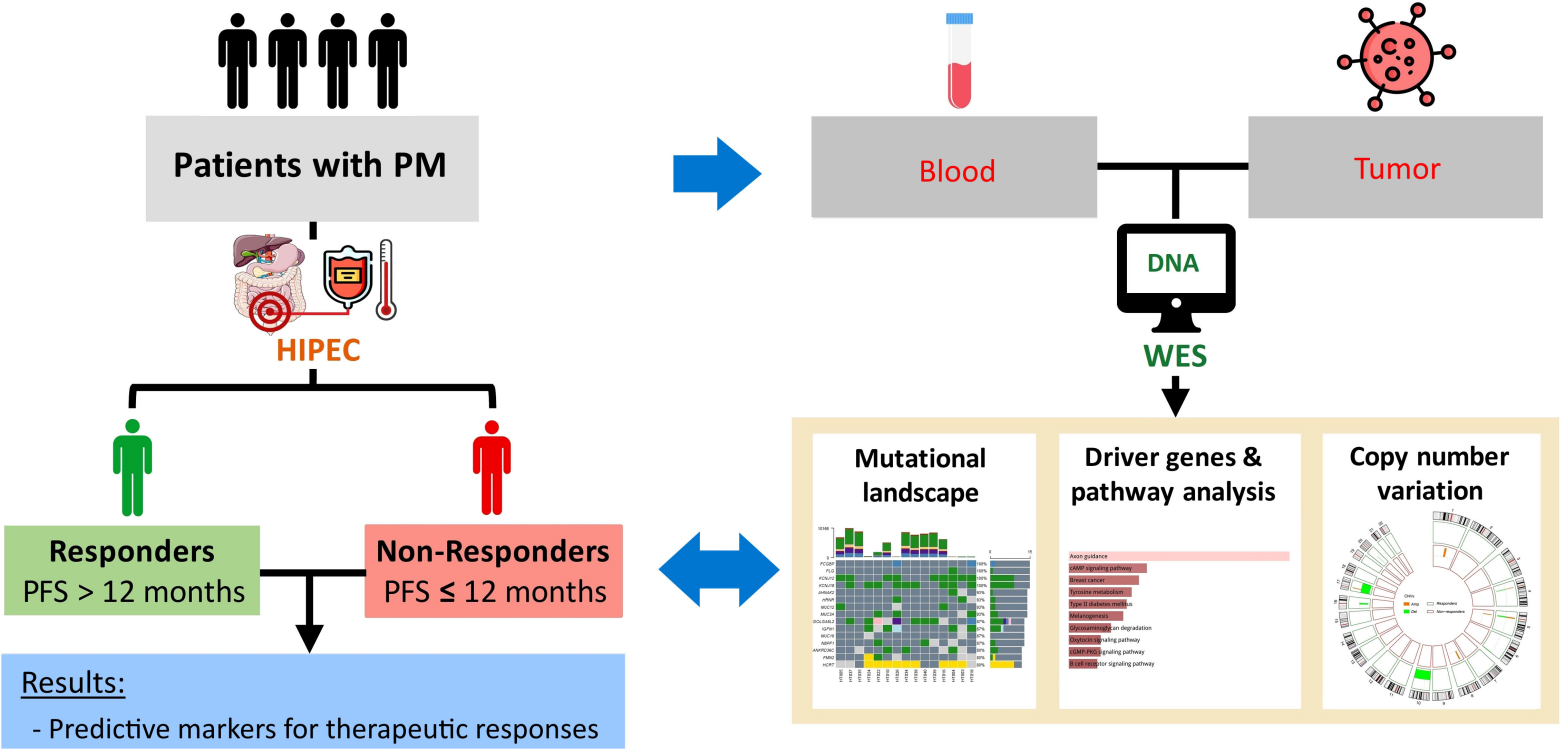
Study design.

This study was conducted with the approval of the Joint Institutional Review Board of Taipei Medical University (approval no. N201807067). The study design is demonstrated in **Figure 1**. Patients diagnosed with AJCC (American Joint Committee on Cancer) stage IV peritoneal metastasis based on computerized tomography (CT) scans, no comorbidities such as heart disease, epilepsy, severe kidney, or liver dysfunction, aged 20–75 years, and with a body weight of 50–100 kilograms were recruited for this study. All eligible patients have signed informed consent forms. After surgery, patients with at least 12 months of follow-up were stratified into two groups based on the 12-month progression-free survival (PFS) (or early recurrence): responders and non-responders.

PFS was defined as the time interval from CRS/HIPEC to the first occurrence of the following events: local or systemic recurrence evaluated by imaging assessment and/or histological confirmation by a laparotomy/laparoscopy; and death from any cause. OS was also investigated from the date of surgery to the date of death from any cause or censored on the date of last follow-up for alive patients.

### 2.2 HIPEC procedure and specimen collection

HIPEC was performed with closed technique for 1 to 2 hours. The PerformerHT (RanD, Medolla, Italy) was used to assure the target temperature ranging from 42.0 to 43.0°C. Chemotherapeutic agents were used in HIPEC as monotherapy or in combination according to primary cancer types, including mitomycin C, docetaxel, cisplatin, and others (etoposide, doxorubicin, mesna, Ifosfamide).

Tumor and matched peripheral blood samples of all patients were collected prior to the HIPEC regimen in the operating room. The collected tumor tissues were stored in RNAlater and kept in liquid nitrogen. The tumor tissues were ground with tissue homogenizers for DNA isolation. Peripheral blood mononuclear cells (PBMCs) were extracted from peripheral blood using Ficoll. DNA was isolated from the tumor tissue and PBMCs with Allprep DNA/RNA kit (Qiagen) according to the manufacturer’s protocol. The DNA concentration per sample was assessed by absorbance measures using the NanoDrop Spectrophotometer (Thermo Scientific, Waltham, MA, USA) and by fluorometric method with the Qubit 2.0 Fluorometer (Thermo Scientific).

### 2.3 Whole exome sequencing and bioinformatic analysis

WES was performed with the Illumina NovaSeq 6000 platform at 150-bp pair-end reads. The average sequencing coverage was 200× for the samples. To assess raw sequencing reads, quality control was performed using FastQC and trimming was performed using Trimmomatic. The Genome Analysis Toolkit version 4.0 (GATK4) was applied for variant calling according to the GATK best practice (28).

The processed reads were mapped and aligned to the reference human genome (GRCh38/hg38) using Burrows-Wheeler Aligner (BWA) (29). BAM files were sorted using SAMtools; polymerase chain reaction (PCR) duplicates were omitted with MarkDuplicates (Picard). Somatic variants (single nucleotide variants (SNVs) and insertions/deletions (INDELs)) were called using GATK4 Mutect2. Subsequently, Ensembl’s Variant Effect Predictor (version 104.3) was used to annotate the variants (30).

The tumor mutational burden (TMB) was calculated as the total number of non-synonymous somatic mutations per mega-base (Mb) of WES data. Mutational data were then analyzed and visualized using the Maftools package (31). Driver genes were identified from the oncodriveCLUST pipeline integrated in the Maftools package. This tool takes into account genes with mutations that are clustered in particular regions of the protein, which are potentially functionally deleterious (32). Kyoto Encyclopedia of Genes and Genomes (KEGG) pathway analysis was subsequently carried out using the web-based tool, Enrichr, for this gene list (33).

### 2.4 Copy number variation analysis

To call the allele-specific copy number for each sample, BAM files were subjected to the Sequenza pipeline, and the GRCh38/hg38 human reference genome assembly was used for annotation (34). Segmentation output files from Sequenza were used as the input for Genome Identification of Significant Targets in Cancer (GISTIC) version 2.0 to detect CNV events at gene level and identify regions with significant amplification or deletion in each cohort (i.e., responders and non-responders) (35). To avoid gender bias, the X and Y chromosomes were excluded from the CNV analysis. Significantly recurrent focal CNV regions in each cohort were determined by GISTIC 2.0 with a false discovery rate (FDR) q-value of < 0.25. The CNV event of a gene was defined as amplification, gain, loss, or deletion based on the GISTIC score.

### 2.5 Statistical analysis

Pre-, peri-, and postoperative clinicopathological data were compared between responders and non-responders. Chi-squared test or Fisher’s exact test was performed for categorical variables. For continuous variables, Student’s *t*-test and Mann Whitney-U test were respectively used for normally distributed and non-normally distributed data. Survival analyses were conducted using the Kaplan-Meier method, and the log-rank test was used to compare survival curves across different groups of interest. A *p* value of < 0.05 was considered statistically significant. All statistical analyses were performed in R version 4.0.5.

## 3 Results

### 3.1 Patient characteristics

In total, 15 patients with PM receiving CRS/HIPEC at Wanfang Hospital (Taipei, Taiwan) were enrolled in this study. Preoperative-, intraoperative-, and postoperative-related clinical data were recorded for analysis (**Tables 1**, **2**). Five patients (33.3%) had primary colorectal cancer (CRC), seven patients (46.7%) had primary gastric cancer, two patients (13.3%) had primary ovarian cancer, and one patient (6.7%) had mesothelioma. This cohort was divided into responders (*n* = 6) and non-responders (*n* = 8) based on their 12-month PFS after CRS/HIPEC. One patient was excluded due to loss of follow-up at three months after CRS/HIPEC. No statistically significant difference was observed between the two groups in any of the patients’ baseline or operative characteristics (**Tables 1**, **2**). As to clinical outcomes, responders achieved a median PFS of 21 (range, 17–33) months and an average OS of 24.83 months, whereas non-responders had a median PFS of 8 (range, 4–11) months and an average OS of 13.12 months (*p* = 0.002 and 0.015, respectively).

**Table 1:** Baseline and preoperative characteristics by response status (*) to CRS/HIPEC.

	Total (n = 15)	Responders (n = 6)	Non-responders (n = 8)	*p-value*
**Gender (%)**				
**Female**	8 (53.3)	4 (66.7)	4 (50.0)	0.627^c^
**Male**	7 (46.7)	2 (33.3)	4 (50.0)	
**Age at inclusion (mean (SD))**	61.67 (9.21)	63.33 (10.25)	60.25 (9.47)	0.571^a^
**BMI (cm/kg^2^) (mean (SD))**	23.28 (4.26)	24.00 (5.38)	22.98 (3.80)	0.684^a^
**ECOG score (median [range])**	1 [1-2]	1 [1]	1 [1-2]	0.471^b^
**Primary cancer (%)**				0.564^c^
**Colorectal cancer**	5 (33.3)	2 (33.3)	2 (25.0)	
**Gastric cancer**	7 (46.7)	2 (33.3)	5 (62.5)	
**Ovarian cancer**	2 (13.3)	1 (16.7)	1 (12.5)	
**Mesothelioma**	1 (6.7)	1 (16.7)	0 (0.0)	
**Time to metastasis (months) (median [range])**	3 [0-54]	7 [0-54]	2 [0-46]	0.947^b^
**PCI score (median [range])**	31 [0-39]	17.5 [0-35]	31 [0-39]	0.3^b^

BMI: Body Mass Index, ECOG: Eastern Cooperative Oncology Group, PCI: peritoneal carcinomatosis index.

^a^: Performed by Student T-test; ^b^: Performed by Mann-Whitney U test; ^c^: Performed by Chi-squared test or Fishers exact test.

(*) Excluded one patient without 12-month PFS data (follow-up < 12 months).

**Table 2:** Operative and postoperative characteristics by response status (*) to CRS/HIPEC.

	Total (n = 15)	Responders (n = 6)	Non-responders (n = 8)	*p-value*
**CC score (median [range])**	2 [0-3]	1 [0-3]	2 [0-3]	0.122^b^
**Operative duration (mins) (mean (SD))**	426.67 (178.28)	481.17 (136.67)	414.12 (200.40)	0.496^a^
**Hospitalization duration (days)** **(median [range])**	11 [5-58]	9 [8-31]	14 [5-58]	0.398 ^b^
**Postoperative complications (%)**	6 (40)	3 (50.0)	3 (37.5)	1^c^
**Pneumonia**	2 (14.3)	0 (0.0)	2 (25.0)	
**UTI**	1 (6.7)	1 (16.7)	0 (0.0)	
**Bowel leakage**	1 (6.7)	1 (16.7)	0 (0.0)	
**Abscess**	1 (6.7)	1 (16.7)	0 (0.0)	
**Intraabdominal infection**	3 (20.0)	2 (33.3)	1 (12.5)	
**Wound infection**	2 (13.3)	0 (0.0)	2 (25.0)	
**Postoperative therapy (%)**	13 (86.7)	4 (66.7)	8 (100)	0.321^c^
**Chemotherapy & Radiotherapy**	4 (26.7)	1 (16.7)	3 (37.5)	
**Chemotherapy**	9 (60.0)	3 (50.0)	5 (62.5)	
**Follow-up (months) (mean (SD))**	17.13 (10.15)	24.83 (6.65)	13.12 (8.76)	**0.015 ^a^**
**PFS (months) (median [range])**	10 [3-33]	21 [17-33]	8 [4-11]	**0.002** ^b^
**OS (months) (mean (SD))**	17.13 (10.15)	24.83 (6.65)	13.12 (8.76)	**0.015** ^a^

CC, completeness of cytoreduction; PFS, progression-free survival; OS, overall survival.

^a^: Performed by Student T-test; ^b^: Performed by Mann-Whitney U test; ^c^: Performed by Chi-squared test or Fishers exact test.

(*) Excluded one patient without 12-month PFS data (follow-up < 12 months). Significant p-values (<0.05) are shown in red.

### 3.2 Somatic mutational profiles in tumor samples of all patients (*n* = 15)

**Figure 2:**
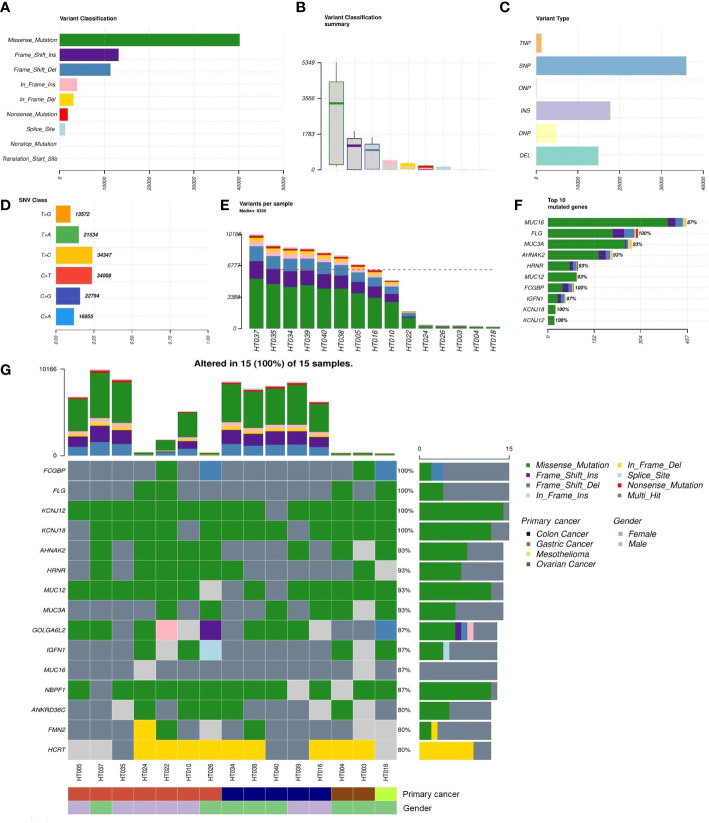
Summary of the non-synonymous mutational landscape (*n* = 15 patients). **(A, B)** Classifications of non-synonymous mutations (i.e., single-nucleotide variants (SNVs) and insertions/deletions (INDELs)). **(C)** Types of non-synonymous mutations. **(D)** Frequencies of transition and transversion events in SNVs. **(E)** Total mutational loads in each tumor sample. **(F)** The top 10 genes with the highest rate of mutation. **(G)** Oncoplot showing the top 15 most frequently mutated genes with detailed annotations in each tumor sample.

There were 15 tumor and matched blood samples obtained prior to the HIPEC regimen for WES. Missense mutations were the highest among the mutational classifications (**Figures 2A, B**), and SNVs dominated all mutational types (**Figure 2C**). The T>C and C>T transitions constituted the highest proportion of SNVs, at approximately 50% of events (**Figure 2D**). Overall, the median total number of mutations per tumor sample was 6350 (range, 253–10,166) (**Figure 2E**). The top 10 genes with the highest rate of mutation were *MUC16* (87%), *FLG* (100%), *MUC3A* (93%), *AHNAK2* (93%), *HRNR* (93%), *MUC12* (93%), *FCGBP* (100%), *IGFN1* (87%), *KCNJ18* (100%), and *KCNJ12* (100%) (**Figure 2F**). Alterations in *FCGBP*, *FLG*, *KCNJ12*, and *KCNJ18* were observed in all tumor samples. Detailed classifications of variants harbored in the top 15 mutated genes are presented in a waterfall plot (**Figure 2G**).

### 3.3 Driver genes and pathway analysis across all tumor samples (*n* = 15)

**Figure 3:**
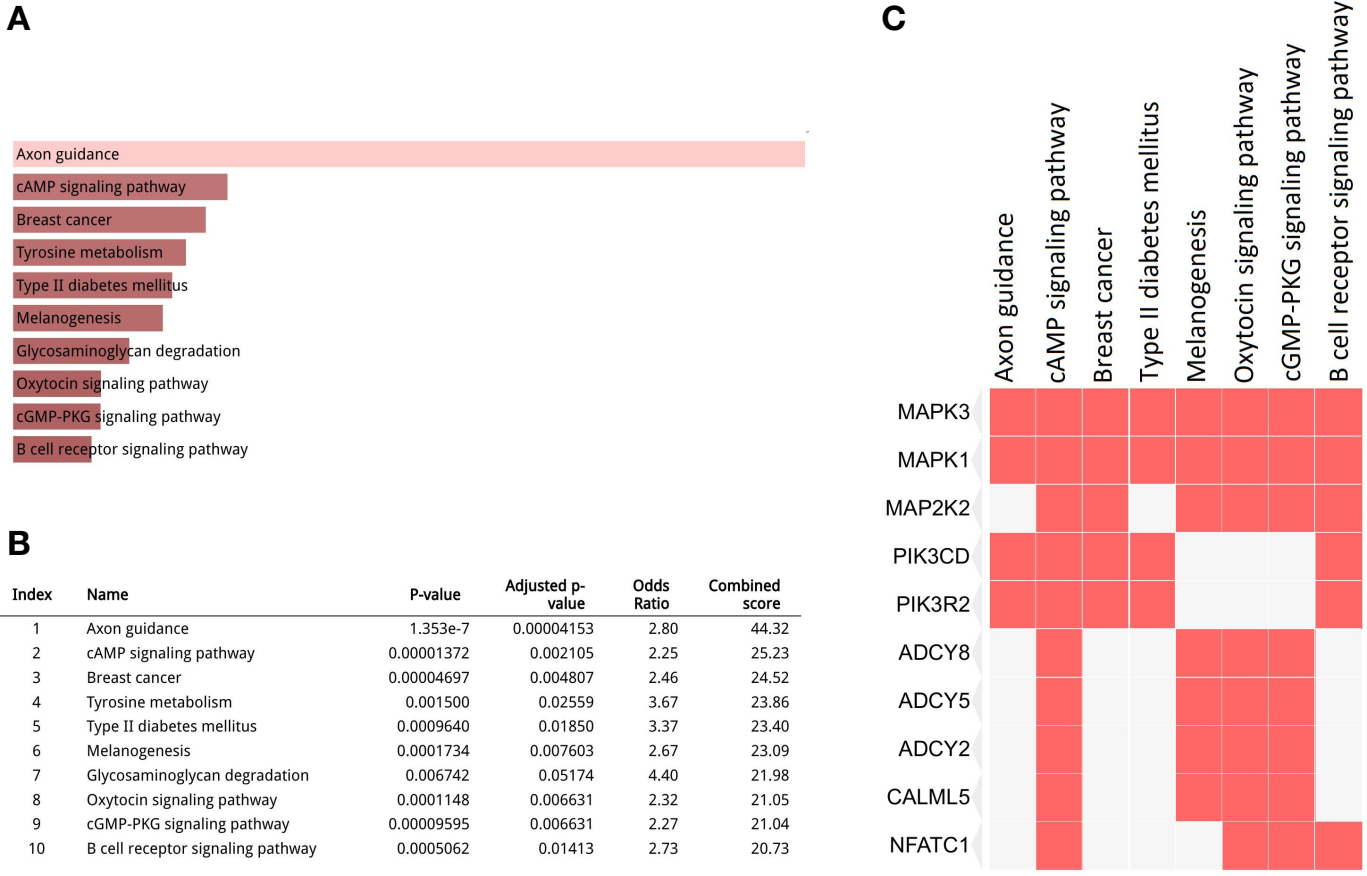
Kyoto Encyclopedia of Genes and Genomes (KEGG) pathway analysis for the driver gene list. **(A, B)** Top 10 enriched pathways (*p* < 0.05, false discovery rate (FDR) < 0.05); **(C)** Clustergram representing genes involved in the enriched pathways.

In total, 1904 potential driver genes were annotated in this cohort using OncodriveCLUST (*p* < 0.05, FDR < 0.05) (**Table S1**). These genes were used as input for the functional gene enrichment analysis (i.e., the KEGG pathway analysis) in the Enrichr web-based tool, which revealed significantly enriched pathways related to signaling, metabolism, tumorigenesis, and immune response (**Figures 3A, B**). Among those, ‘axon guidance’ was a significantly enriched pathway in this cohort. Molecules involved in this pathway are listed in **Table S2**. As shown in the **Figure 3C**, mitogen-activated protein kinase 1 (*MAPK1*) and *MAPK3* were the most frequently clustered genes in the top annotated enriched pathways.

### 3.4 Comparisons of tumor molecular characteristics between responders and non-responders

We further compared the TMB between responders and non-responders. Results revealed higher TMB in pretreatment tumors of non-responders; however, the difference did not reach statistical significance (median TMB of 133.04 mutations/Mb vs. 71.14 mutations/Mb, *p* = 0.4316) (**Figure S1**). Likewise, while the total number of non-synonymous (ns)SNVs was relatively higher in non-responders than in responders (median of 3147 vs. 1892, *p* = 0.4908), the impact of nsSNV loads on patient outcomes was not clearly established (**Figure S2**).

**Figure 4:**
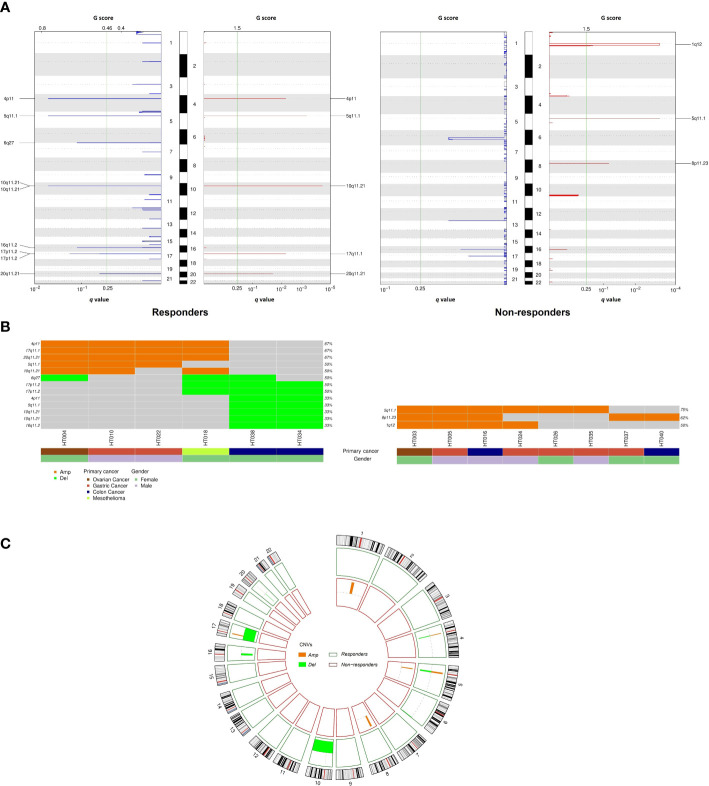
Comparison of copy number variation between responders and non-responders. **(A)** Amplifications and deletion peaks identified by GISTIC2.0 (q < 0.25); **(B)** Recurrent focal amplification and deletion regions detected for each tumor; **(C)** Circos plot summarizing copy number variation distribution in the two cohorts.

In the CNV analysis, fewer amplification than deletion events were observed in responders at the chromosome-arm level, while there were only amplified chromosomal events in non-responders (**Figures 4A, C**). Specifically, gains of 4p, 5q, 10q, 17q, 20q, and gains of 1q, 5q, and 8p were respectively reported for responders and non-responders. Meanwhile, chromosomal losses of 4p, 5q, 6q, 10q, 16q, 17p, and 20q were exclusively observed in responders. In total, eight recurrent focal amplification and nine deletion peaks were also identified across cohorts (*q* value of < 0.25) (**Figure 4B**). Among them, amplification at 4p11 (67%), 17q11.1 (67%), and 20q11.21 (67%) and deletion at 6q27 (50%) and 17p11.2 (50%) were the most frequent events in responders. On the other hand, amplification of 5q11.1 (75%) was mostly observed in non-responders. The amplification and deletion events at gene level are summarized in **Table S3**.

### 3.5 Mutations of *AGAP5* associated with prognostic significance

**Figure 5:**
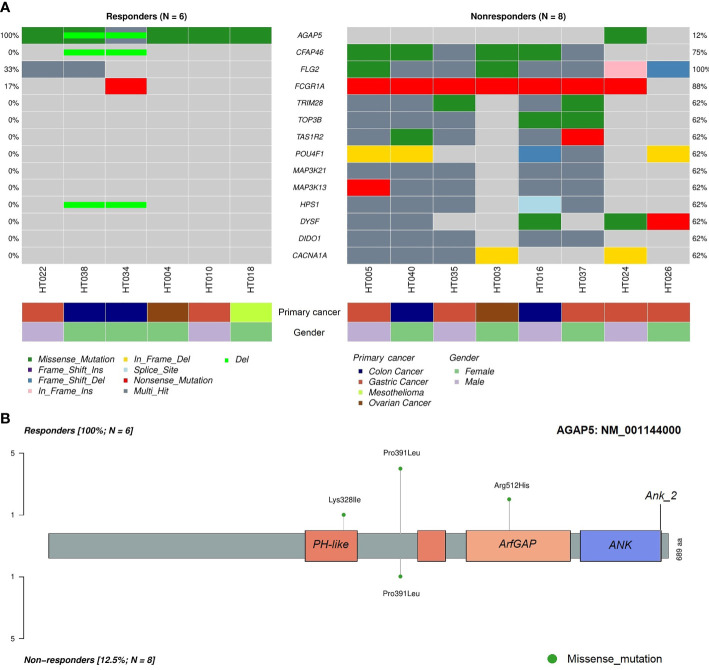
Differentially mutated genes in tumor samples between responders and non-responders. **(A)** Differentially mutated genes between the two cohorts with frequencies and classifications of mutations. **(B)** Lollipop plot demonstrating mutated amino acids on different functional domains of *AGAP5*. ANK, ankyrin repeat; PH, pleckstrin homology.

**Figure 6:**
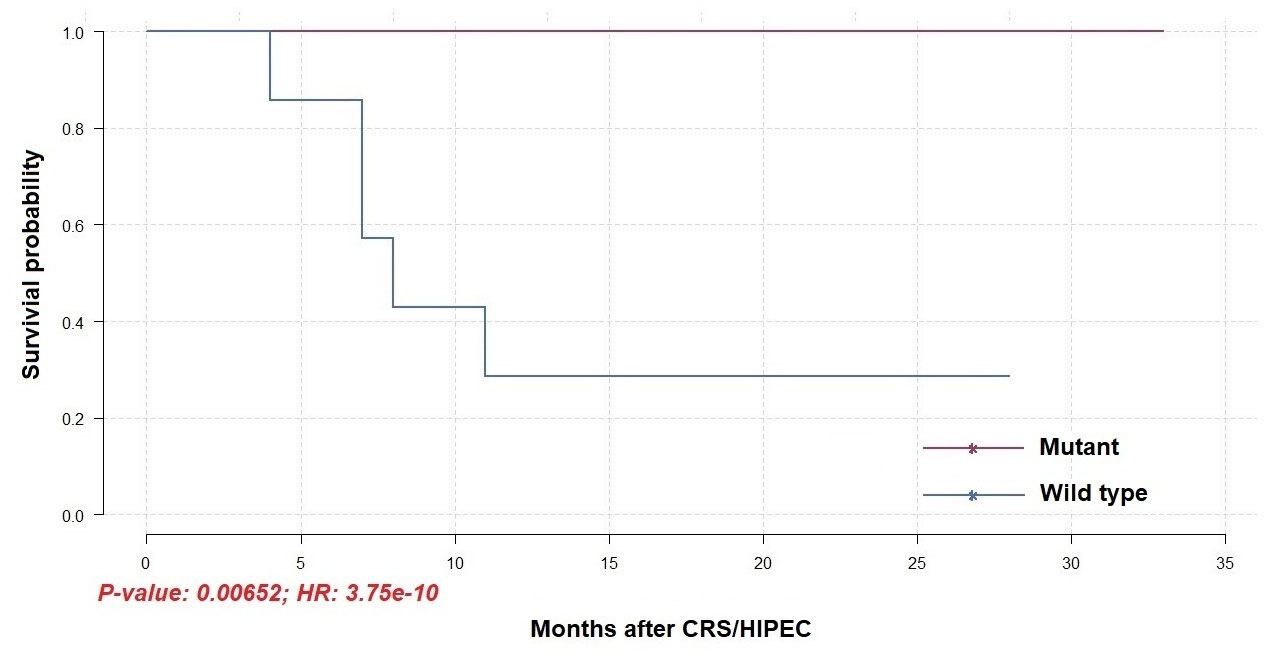
Overall survival of patients receiving CRS/HIPEC with or without a mutated *AGAP5* gene (N = 14).

To identify differentially mutated genes, we conducted Fisher’s exact test for all genes with alterations between responders and non-responders. Fourteen genes were recurrently and preferentially mutated in either responders or non-responders (*p* < 0.05) (**Figure 5A**). Among those, *CFAP46*, *TRIM28*, *TOP3B*, *TAS1R2*, *POU4F1*, *MAP3K21*, *MAP3K13*, *HPS1*, *DYSF*, *DIDO1*, and *CACNA1A* were found as unique in tumors from non-responders. Notably, *AGAP5* was the top differentially mutated gene, with missense mutations reported in all responding tumors (100%) and one of eight samples in non-responders (12.5%). The pattern of missense mutations on different functional domains of *AGAP5* is demonstrated with a lollipop plot (**Figure 5B**). In the survival analysis, mutated *AGAP5* was significantly correlated with better OS following CRS/HIPEC compared to the wild-type gene (*p* = 0.00652, hazard ratio (HR) = 3.75 × 10^-10^) (**Figure 6**). Besides *AGAP5*, results of the survival analyses for the remaining genes are shown in **Figure S3**. No significant associations were identified.

## 4 Discussion

In this study, molecular signatures from WES may facilitate clinical decision-making for PM management. First, alterations in *FCGBP, FLG, KCNJ12*, and *KCNJ18* were observed in all tumor samples, regardless of the responsiveness towards HIPEC. *FCGBP* is involved in the intestinal immune defense against inflammation and carcinogenesis. Reduced expression of *FCGBP* was frequently observed in colorectal adenoma and CRC (36). *FLG* acts as a defense line (i.e., establishment of a skin barrier (GO:0061436)) that exhibits mutations in several cancer types (e.g., non-melanoma skin cancer, CRC, cervical cancer, and prostate cancer) (37), whereas *KCNJ12* and *KCNJ18* are involved in regulating transmembrane transport (GO:0034762), which is important in cell proliferation, differentiation, and apoptosis (38). Acquired mutations may imply impaired functions of these genes in primary tumorigenesis as well as the development of PM. 

Importantly, ‘axon guidance’ was annotated as the most important pathway in our cohort. The ‘axon guidance’ pathway modulates a variety of key biological functions including cell survival, proliferation, differentiation, migration, and invasion in different cancer types (39). Dysregulation of axon guidance molecules has frequently been reported in cancers. *EPHB6* was down-regulated in several cancer types such as metastatic lung cancer, melanoma, and CRC (40–42), while high expression of *SEMA4D* promoted angiogenesis in many tumors (43, 44). Pepinemab, an anti-SEMA4D has been developed and tested in several solid tumors (45, 46). An early phase clinical trial has demonstrated that Pepinemab combined with other immune checkpoint inhibitors was well-tolerated and showed anti-tumor activity in immune-resistant tumors (46). Recently, Yu et al. reported that suppression of *ERBB2* (Erb-B2 Receptor Tyrosine Kinase 2) was observed with the negative regulatory activity on MAPK1/MAPK3 signaling pathway that led to the hinderance of tumor progression in ovarian cancer cells (47). Taken together, these findings revealed potential therapeutic targets for PM.

Our results indicated that TMB was higher in non-responders than that in responders, which is consistent with a previous finding of colorectal PM treated with CRS/HIPEC (48). Conversely, the correlation between high TMB and better responses to HIPEC was reported in gastric PM patients (49). The inconsistent results might be due to the tumor heterogeneity. In addition, the CNV analysis revealed targets with prognostic potential for therapeutic response and survival outcomes. For example, *ABCC3* (17p11.2) and *ABCC2* (10q11.21) were found to be deleted in responders. These ATP-binding cassette (ABC) transporters regulate the efflux of drugs from cancer cells; therefore, deletions in these gene loci were previously described as producing favorable responses to neoadjuvant chemotherapy (50). In contrast, five of eight non-responding samples exhibited amplified *ANK1* and *FGFR1* in the 8p11.23 region. High expression of *ANK1* and *FGFR1* are associated with poor outcomes in several cancer types (51, 52).

Here, we identified 11 exclusively mutated genes in non-responders, namely *CFAP46*, *TRIM28*, *TOP3B*, *TAS1R2*, *POU4F1*, *MAP3K21*, *MAP3K13*, *HPS1*, *DYSF*, *DIDO1*, and *CACNA1A*. Importantly, we found that mutated *AGAP5* was observed in all responders and was associated with increased OS after CRS/HIPEC. In addition, deletion of *AGAP5* was also detected in responders HT034 and HT038 (**Figure 5A**).

*AGAP5* is a protein-coding gene with the function of a GTPase activity activator. *AGAP5* consists of an ADP-ribosylation factor (Arf) GAP domain, ankyrin repeat (ANK) domains, and pleckstrin homology (PH) domain 5 (**Figure 5B**). The PH domain is a critical protein-binding site for catalyzing GTPase activity, and the PH-Arf GAP domain interaction is necessary to activate AGAP function (53). Arfs are proteins that belong to a subfamily of Ras small GTPases without intrinsic GTPase activities. Arfs are activated by GTP exchange factors (Arf GEFs) that assist the conversion of GDP to GTP and are terminated by GTPase-activating proteins (Arf GAPs) (54). In addition, a growing body of evidence suggests the functions of Arf GAPs through an Arf-independent manner (55–57). Under normal physiological circumstances, Arf proteins play important roles in regulating key cellular functions, including membrane trafficking, lipid metabolism, energy utility, cell motility, mitosis, apoptosis, and transcription (54, 58). Upregulation of Arf1, Arf4, and Arf6 were found in breast, gastric, prostate, or lung cancer. Furthermore, overexpression of Arf GAPs (i.e., *AGAP1* and *AGAP2*) was identified in different cancer types such as breast, colon, lung, ovarian, and hepatocellular carcinoma (57). *AGAP2* with regulatory activity on Arf1 and Arf5 enhances cancer cell survival, migration, and invasion in glioblastoma (59). Amplified *AGAP2* and *ASAP1* were associated with impaired OS and progression-free survival in uveal melanoma (60).

The limitation of this study is the small sample size. To establish a better signature to predict the therapeutic outcomes, more clinical samples for further analysis are needed. In addition, our cohort comprised patients with PM derived from different origins (i.e., CRC, gastric cancer, ovarian cancer, and mesothelioma); thus, sub-group analyses according to primary cancer types should be considered to generate more stringent conclusions. Despite these shortcomings, we successfully identified potential shared genetic markers with prognostic value for CRS/HIPEC. These exploratory findings may provide a rationale for clinical decision-making. 

In conclusion, we uncovered the molecular characteristics from PM patients and reported a list of driver genes as well as enriched signaling pathways. The results might be helpful for further drug discovery. Importantly, *AGAP5* was found to be significantly associated with the better OS. This finding is helpful to detect the good responders after CRS/HIPEC. In summary, our study provides a tailor prognostic signature for CRS/HIPEC therapy.

## Data availability statement

The datasets presented in this study can be found in online repositories. The names of the repository/repositories and accession number(s) can be found below: https://www.ncbi.nlm.nih.gov/bioproject/PRJNA891798, accession number PRJNA891798.

## Ethics statement

The studies involving human participants were reviewed and approved by The Joint Institutional Review Board of Taipei Medical University (approval no. N201807067). The patients/participants provided their written informed consent to participate in this study.

## Author contributions

Q-AN, W-HC, Y-TT, M-CH and W-CC conceptualized, conceived, and designed the study. Y-TT, M-CH performed clinical diagnosis and sample collection. Q-AN, C-MC, W-TL prepared samples for sequencing. Q-AN, W-HC, W-CC analyzed data. Q-AN, W-HC, Y-TT, M-CH and W-CC interpreted results. Q-AN, W-HC wrote the original draft. Y-TT, M-CH and W-CC provided the funding. All authors contributed to the article and approved the submitted version.

## Supplementary material

Supplementary Table 1 List of driver genes.

Supplementary Table 2 KEGG pathway analysis of driver genes.

Supplementary Table 3 Genes with an amplification or deletion event.
