# Supplementary figures and images for: Genetic alterations in peritoneal metastatic tumors predicted the outcomes for hyperthermic intraperitoneal chemotherapy
Source: Front Oncol. 2023 Apr 26;13:1054406. doi: 10.3389/fonc.2023.1054406 (PMC10170308; doi:10.3389/fonc.2023.1054406)

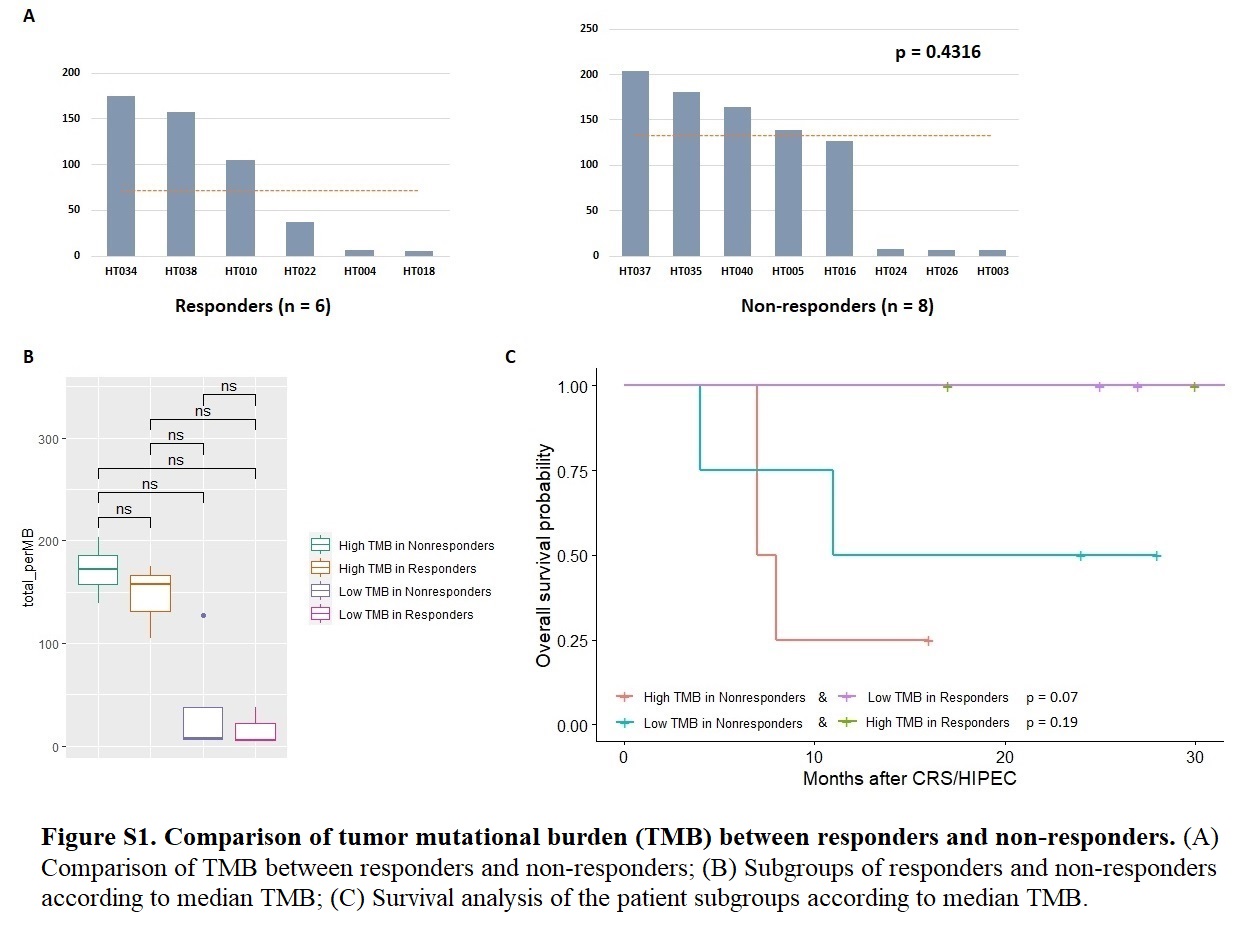

Supplement: Supplementary Table 1 — List of driver genes. [file Image_1.jpeg]

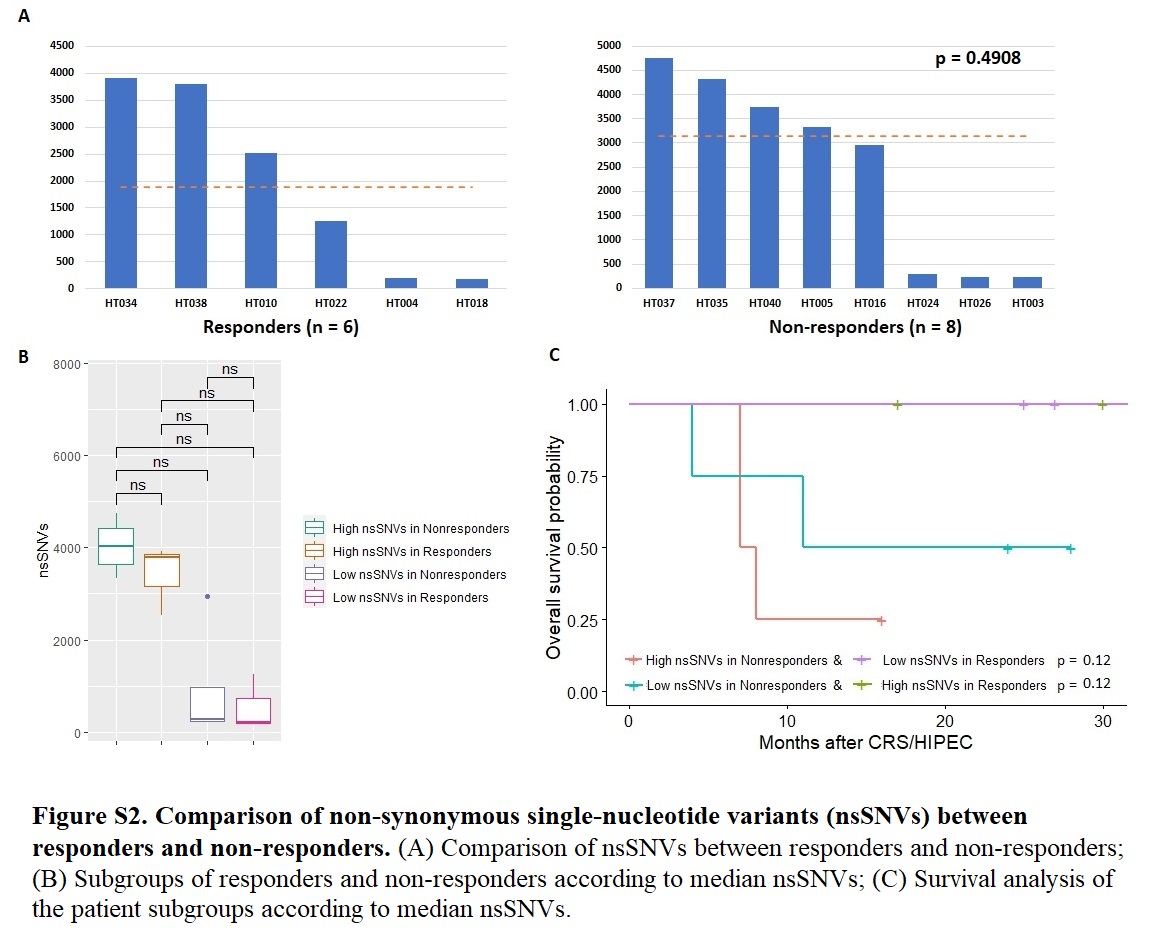

Supplement: Supplementary Table 2 — KEGG pathway analysis of driver genes. [file Image_2.jpeg]

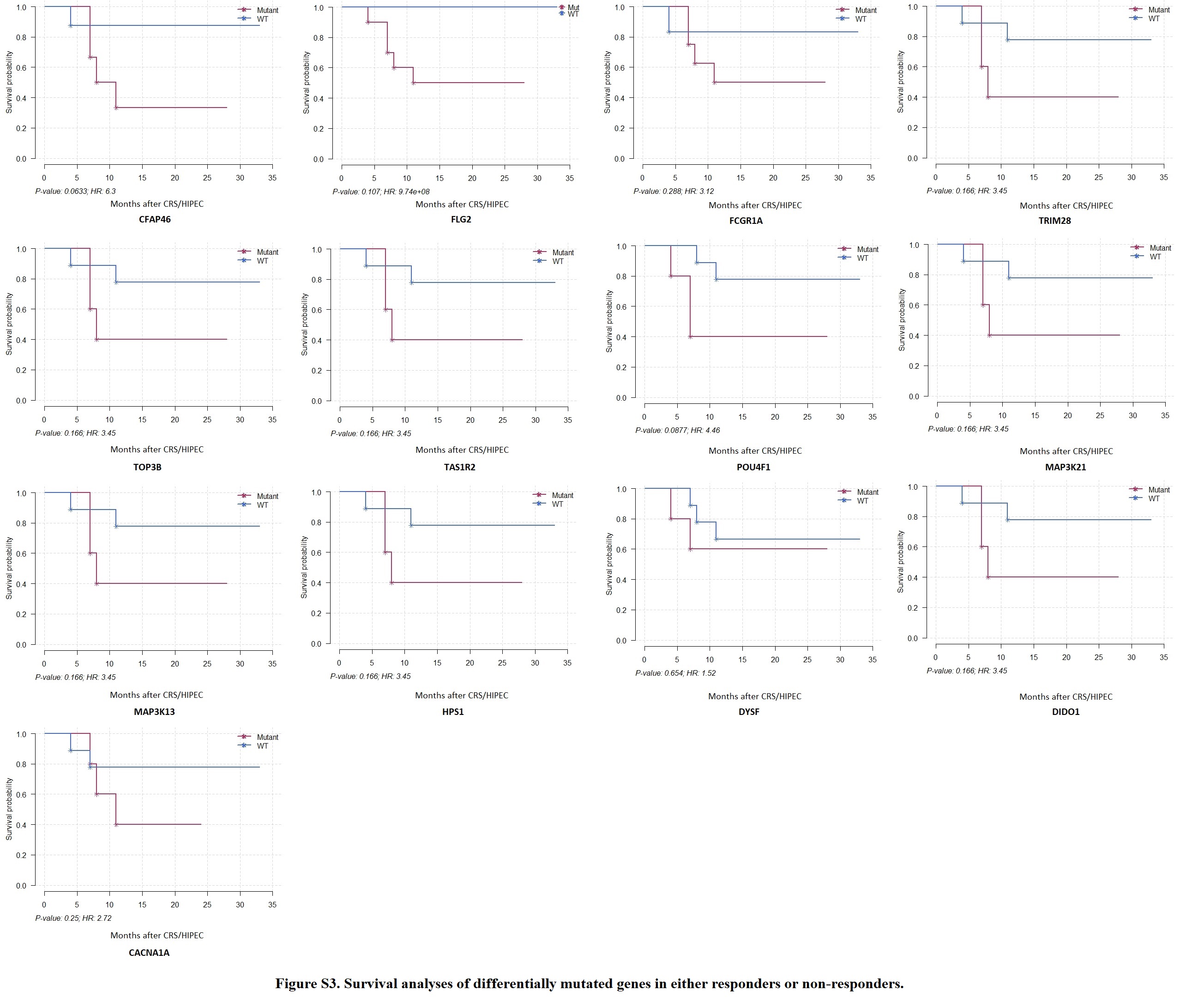

Supplement: Supplementary Table 3 — Genes with an amplification or deletion event. [file Image_3.jpeg]
